# Shifts in potential geographical distribution of *Pterocarya stenoptera* under climate change scenarios in China

**DOI:** 10.1002/ece3.6236

**Published:** 2020-04-06

**Authors:** Keliang Zhang, Huina Liu, Haolei Pan, Wenhao Shi, Yi Zhao, Silei Li, Junchi Liu, Jun Tao

**Affiliations:** ^1^ Jiangsu Key Laboratory of Crop Genetics and Physiology College of Horticulture and Plant Protection Yangzhou University Yangzhou China

**Keywords:** climate change, ecologic niche modeling, GARP, Maxent, potential suitable habitat, *Pterocarya stenoptera*

## Abstract

Climate change poses a serious threat to biodiversity. Predicting the effects of climate change on the distribution of a species' habitat can help humans address the potential threats which may change the scope and distribution of species. *Pterocarya stenoptera* is a common fast‐growing tree species often used in the ecological restoration of riverbanks and alpine forests in central and eastern China. Until now, the characteristics of the distribution of this species' habitat are poorly known as are the environmental factors that influence its preferred habitat. In the present study, the Maximum Entropy Modeling (Maxent) algorithm and the Genetic Algorithm for Ruleset Production (GARP) were used to establish the models for the potential distribution of this species by selecting 236 sites with known occurrences and 14 environmental variables. The results indicate that both models have good predictive power. Minimum temperature of coldest month (Bio6), mean temperature of warmest quarter (Bio10), annual precipitation (Bio12), and precipitation of driest month (Bio14) were important environmental variables influencing the prediction of the Maxent model. According to the models, the temperate and subtropical regions of eastern China had high environmental suitability for this species, where the species had been recorded. Under each climate change scenario, climatic suitability of the existing range of this species increased, and its climatic niche expanded geographically to the north and higher elevation. GARP predicted a more conservative expansion. The projected spatial and temporal patterns of *P. stenoptera* can provide reference for the development of forest management and protection strategies.

## Graphical abstract

Minimum temperature of coldest month (Bio6), mean temperature of warmest quarter (Bio10), annual precipitation (Bio12), and precipitation of driest month (Bio14) were important environmental variables influencing the prediction of the Maxent model. Under each climate change scenario, climatic suitability of the existing range of this species increased, and its climatic niche expanded geographically toward farther north and higher elevation. The GARP predicted a more conservative expansion.

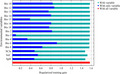

## 1 INTRODUCTION

Climate is one of the major factors contributing to the large‐scale distribution of species (Stocker et al., 2013). Global climate change has affected the distribution and abundance of numerous species in the past few decades (Zhang, Yao, Meng, & Tao, 2018) and also contributes to the extinction of species (Thuiller, Lavorel, & Araujo, 2005). The potential threats that may change the scope and distribution of species can be identified by predicting the effects of climate change on the landscape‐scale spatial distribution of habitat for individual species. Therefore, high‐quality distributional data play an important role in setting priorities and implementing effective protection actions (Brooks, M'Lot, & McLachlan, 2006). However, the Wallacean shortfall, i.e. the deficiency of biogeographical information, often hinders various conservation actions (Bini, Diniz‐Filho, Rangel, Bastos, & Pinto, 2006).

Ecological niche modeling (ENM) provides an important tool that can be used to fill this gap and can also promote the study of ecology, conservation, and evolution (Elith, Kearney, & Phillips, 2010; Renner & Warton, 2013; Solano & Feria, 2007). This type of modeling has been widely used in the study of habitat fragmentation (Austin, 2007; Thuiller, Araujo, & Lavorel, 2004), how climate change affects biodiversity (Heikkinen et al., 2006), the development of conservation plans for rare species (Marage, Garraud, & Rameau, 2008; Zhang et al., 2018), and range expansion of invasive species (Peterson, Papes, & Eaton, 2007; Reino et al., 2009; Welk, 2004). A variety of ENMs, such as CLIMEX, maximum entropy (Maxent), genetic algorithm for rule‐set production (GARP), ecological niche factor analyses, and bioclimatic prediction systems, have been employed for the prediction of the distribution areas, ecological responses, and ecological requirements of various species. In general, these tools are different in the predictors used (physiological constraints in a mechanistic approach or climatic empirical approach) and species records (presence‐only or presence/absence) (Mac Nally, 2000; Peterson et al., 2007).

Maxent and GARP provide two commonly used niche‐based modeling methods that use presence‐background, and both have been used to predict the spatial distribution of species at different scales (Larson, Olden, & Usio, 2010; Phillips, Anderson, & Schapire, 2006; Stockwell & Noble, 1992; VanDerWal, Shoo, Graham, & Williams, 2009). Maxent is a generalized linear model; it produces models by finding the distribution closest to uniform (maximum entropy) constrained by the input of environmental variables (Phillips et al., 2006). In contrast, GARP, a superset of modeling algorithms, searches for nonrandom relationships between ecological conditions and species occurrence data at sites. It constructs a set of rules to describe a species' ecological niche defined as a set of conditions where a species can thrive in the environment (Elith et al., 2010; Renner & Warton, 2013; Stockwell & Noble, 1992). The differences between the two algorithms in their procedures and rationales will result in different performance (Hernandez, Graham, Master, & Albert, 2006). Previous studies indicate that the potential distribution generated by GARP is wider than that produced by Maxent (Hernandez et al., 2006).

**FIGURE 1:**
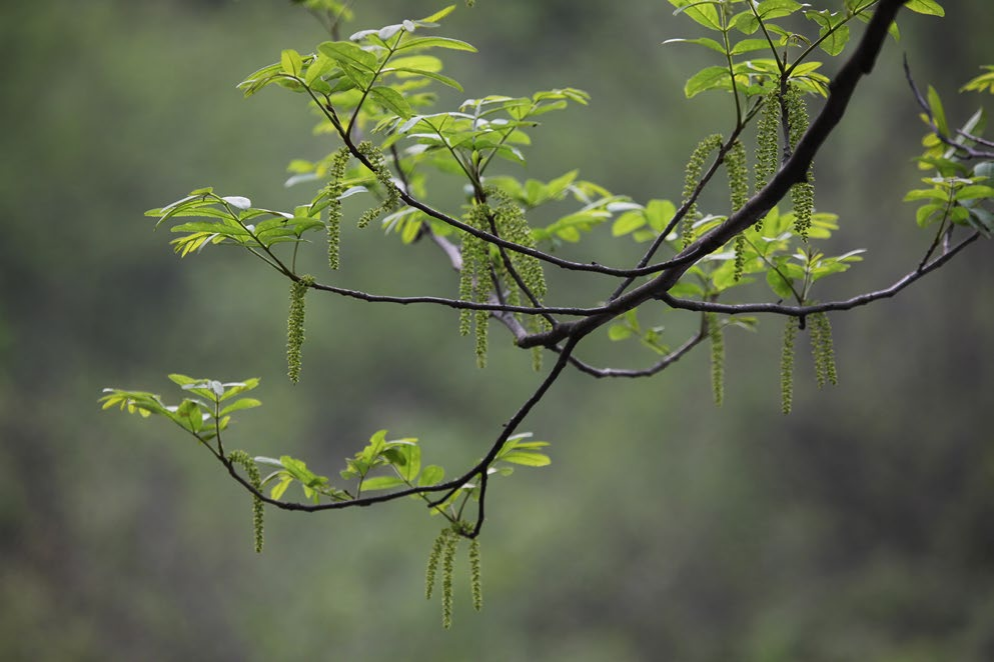
Photograph of *Pterocarya stenoptera*

*Pterocarya stenoptera* C. DC. (Juglandaceae), a species endemic to China, is a deciduous broad‐leaved tree (Figure 1) that grows in forests along wet hillside land or along streams at elevations below 1,500 m above sea level (Lu, Stone, & Grauke, 1999). With a wide distribution in warm temperate and subtropical zones of China, the species can grow to a height of 20 m in the first 5 years and grows on acidic and slightly alkaline soil (Lu et al., 1999). Also, it can tolerate long‐term flooding. Yang, Li, Li, Schneider, and Lamberts (2013) reported that plants of *P. stenoptera* could survive in continuous submergence or inundation for 12 months. When compared with unplanted soils, the presence of *P. stenoptera* seedlings resulted in a significant increase in total nitrogen and total phosphorus in soil (Yang et al., 2013). In addition, this species has shown resistance to various diseases and pests that typically threaten walnut trees (Pan, 2009). Therefore, it has been commonly planted during ecological restoration projects along riverbanks and in alpine forested areas in the eastern and central parts of China (Pan, 2009). However, researchers know little about the potential geographical distribution of the species and the environmental factors that affect the suitability of habitat for this taxon. Knowing how climate change will affect habitat suitable for *P. stenoptera* is an important issue given the economic and ecological significance of the species.

To determine the potential geographical distribution and the environmental variables influencing habitat suitability for this species, we used Maxent and GARP modeling to predict the future distribution of *P. stenoptera.* The objectives of this study were (a) to model this species' potential geographical distribution; (b) to identify the most important environmental factors shaping the distribution of *P. stenoptera*; and (c) to discuss the variations in the distribution of suitable habitat under climate change. The results will allow researchers to identify the future suitable habitat and help in the use, management, and cultivation of *P. stenoptera*.

## 2 MATERIAL AND METHODS

### 2.1 Species occurrence data

**FIGURE 2:**
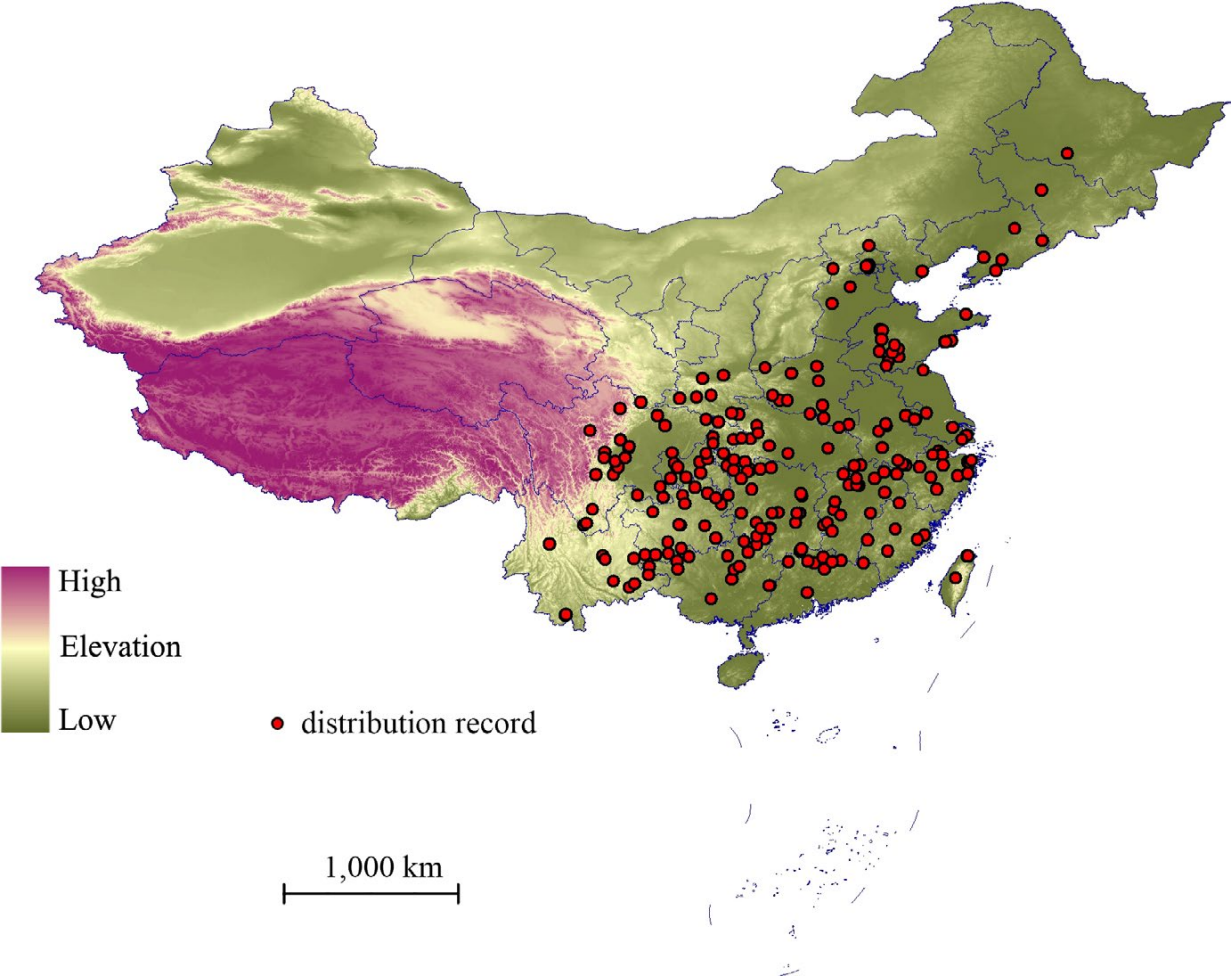
Distribution records of *Pterocarya stenoptera* in China. Outlines of provinces and other administrative areas are shown

Species point locality data were collected from the following online herbaria databases: Tropicos (http://www.tropicos.org/), the Global Biodiversity Information Facility (http://www.gbif.org) and the Chinese Virtual Herbarium (http://v5.cvh.org.cn/) database; the latter holds the plant distribution records of the main herbaria of China. Some results from other field survey reports and scientific research literature (Li, Wei, Lü, & Zhang, 2010; Lu et al., 1999; Pan, 2009; Wang, Xu, Li, Zhao, & Zhang, 2018; Yang et al., 2013) were also included. The analysis excluded imprecise locations when no exact geo‐coordinates exist in the occurrence records. The longitude and latitude of any specimens in the Chinese Virtual Herbarium that provided only the village location were determined using Google Earth (http://ditu.google.cn/) (Wei, Wang, Hou, Wang, & Wu, 2018). After deleting duplicate points, spatial filtering was carried out for the remaining data points. Therefore, only one point was mapped in each 1.0 × 1.0 km grid cell. A total of 236 unique geo‐referenced occurrence records were used (Figure 2).

### 2.2 Environmental Data

Soil variables are important factors controlling plant distribution; they change over relatively short timescales based on direct and indirect effects caused by anthropogenic climate change (Richter & Markewitz, 2001). As a result, soil and climate variables should both be included in modeling. Nevertheless, the expected changes in soil variables under future climate scenarios are not generally available for China. Therefore, Stanton, Pearson, Horning, Ersts, and Reşit Akçakaya (2012) have suggested better results could be achieved by combining important static variables for which reliable projections are unavailable with dynamic climate variables whose future values have been predicted by general circulation models; this will produce a better result than excluding static variables. So, we used both dynamic climatic variables and static environmental variables in our models. Nineteen dynamic bioclimatic variables (11 temperature and eight precipitation metrics) were acquired from WorldClim (www.worldclim.org/bioclim; Hijmans, Cameron, Parra, Jones, & Jarvis, 2005). Three static soil variables, that is, soil class (SCla), soil organic carbon (SC), and soil pH (SpH), were acquired from the Resource and Environment Data Cloud Platform (http://www.resdc.cn/Default.aspx) for 1984–1995 data.

For future climate scenarios, BCC‐CSM1.1 climate change modeling data under the Representative Concentration Pathways (RCPs) 2.6 and 8.5 proposed by the Intergovernmental Panel on Climate Change were used for the years 2050 and 2070. The BCC‐CSM1.1 is recommended for use in studies of operational short‐term climate prediction and studies of climate change in China [see Wu et al. (2014) and references cited therein]. Scenario RCP 2.6 reflects potential radiative forcing by 2100 compared with the pre‐industrial values of +2.6 W/m^2^ which is optimistic, while RCP 8.5, a more pessimistic scenario, reflects high levels of greenhouse gas emissions, and results in 8.5 W/m^2^ of radiative forcing in 2100 (Hijmans et al., 2005). The three static soil variables remained unchanged for the analysis of ENM under future projected climate conditions. To ensure consistency across all layers, all environmental layers were processed using the same spatial extent, cell size, and WGS84 projection in ArcGIS 10.0. The raster files were projected to an equal‐area grid and a 1.0‐km spatial resolution was used.

Principal component analysis and correlation analyses (Pearson's correlation coefficient) were carried out to minimize the overfitting of the model and decrease the high collinearity. Only one of the highly correlated variables (|*r*| > .90) in each set was retained for further analysis. The variables in the final environmental dataset include precipitation of warmest quarter (Bio18), precipitation of wettest quarter (Bio16), precipitation seasonality (Bio15), precipitation of driest month (Bio14), precipitation of wettest month (Bio13), annual precipitation (Bio12), mean temperature of warmest quarter (Bio10), mean temperature of wettest quarter (Bio8), minimum temperature of coldest month (Bio6), temperature seasonality (Bio4), isothermality (Bio3), and mean diurnal temperature range (Bio2).

### 2.3 Model simulation

Desktop GARP version 1.1.3 (Stockwell & Peters, 1999) and Maxent version 3.3.3 k (Phillips et al., 2006) were used to establish models according to the bioclimatic variables and the species records. For Maxent, 25% of occurrence records were used to test the model and 75% of occurrence records were applied for training. Sampling bias is known for having significant effects on the results of presence‐background distribution models (Elith et al., 2010; VanDerWal et al., 2009). A bias file layer was used to avoid sampling bias in the species occurrence data (Phillips et al., 2009). This file was generated using occurrence point by deriving a Gaussian kernel density map that was rescaled from 1 to 20 based on Elith et al. (2010). The maps were created using the bias file option in Maxent. Recent studies noted that nonoptimal models may result if the default configuration is used so it may not always be appropriate, particularly when a limited number of occurrence records of a species are available. Therefore, we analyzed different regularization multiplier values, finding that the default option performed best. That is, the default option provided the best display of the known distribution of *P. stenoptera* but did not overfit the model [see Merow, Smith, and Silander (2013)]. The number of background points for sampling was limited to 10,000 in the present study. Nevertheless, we also checked that increasing the background points (e.g., 100,000) failed to change the model. Maxent's “fade by clamping” function was used to modify the areas with projections that were affected by clamping. We set the maximum numbers of iterations to 1,000; this allows adequate time for model to reach convergence; 1 × 10^−6^ was selected as the convergence threshold (Deb, Phinn, Butt, & McAlpin, 2017a). In addition, we used the default “autofeatures,” including linear, quadratic, product, threshold, and hinge features (Merow et al., 2013).

Genetic Algorithm for Ruleset Production uses a sets of conditional rules that were developed iteratively for rule selection, evaluation, testing, and incorporation or rejection (Peterson et al., 2007). We used 25% of occurrence records to test the model and 75% of occurrence records for training. The best‐subsets selection procedure (Anderson, Lew, & Peterson, 2003; Phillips et al., 2006) was used with the maximum number of iterations of 1,000, the convergence limit of 0.01, and 20 runs per model. Ten best models were finally selected to produce a single final grid. The best subset selection standards included omission error with the lowest value of 20% and commission error with the default value of 50% (Anderson et al., 2003). Other default parameters were maintained (Anderson et al., 2003).

### 2.4 Model evaluation

The patterns of the Maxent output were explained through the response curves. The significance of each variable for Maxent predictions was examined by carrying out the Jackknife test. The models were evaluated according to true skill statistic (TSS) (Allouche, Tsoar, & Kadmon, 2006), omission rate (OR), and area under the receiver operating characteristic curve (AUC) (Fielding & Bell, 1997). The AUC is a threshold‐independent statistic, ranging from 0 to 1. An AUC value around 0.5 indicates the prediction provided by a distribution model was no better than random, while values around 1 indicate the observed species distribution was well fitted with the predicted distribution of a species. The distribution models with values greater than 0.7 were acceptable. The omission rate (OR) is also a threshold‐independent statistic, and high‐quality models should show zero or low OR [for more details, see Pearson et al. (2006)]. TSS is a statistic dependent on threshold, ranging from −1 to 1 (Allouche et al., 2006). TSS values near 0 or negative indicate the distributions were no better than a random pattern, while the values equal to +1 represent the observed distribution was consistent with the predicted distribution. The TSS, OR and AUC were calculated for the ten models of both algorithms. A one‐tailed Wilcoxon signed‐rank test was used to evaluate the statistical significance of AUC, TSS, and OR values between GARP and Maxent.

## 3 RESULTS

### 3.1 Model evaluation and comparison

The average AUC of models generated by Maxent and by GARP were 0.996 and 0.976, respectively, indicating the good performance of both models (Table 1). According to the results of the one‐tailed Wilcoxon signed‐rank test, AUC and TSS of Maxent were statistically significantly higher than that of GARP, while OR of GARP is statistically significant higher than Maxent (Table 1).

**TABLE 1:** Comparison of AUC, TSS, and OR of GARP and Maxent models

Model	Area under the curve (AUC)	True skill statistic (TSS)	Omission rate (OR)
Maxent	0.996 ± 0.002	0.949 ± 0.029	0.012 ± 0.002
GARP	0.976 ± 0.014	0.915 ± 0.052	0.027 ± 0.008
*p* value^a^	<.001	<.001	.005

^a^ *P* value of the one‐tailed Wilcoxon sign‐ranked test on AUC, TSS, and OR between GARP and Maxent model.

### 3.2 Important environmental variables

**FIGURE 3:**
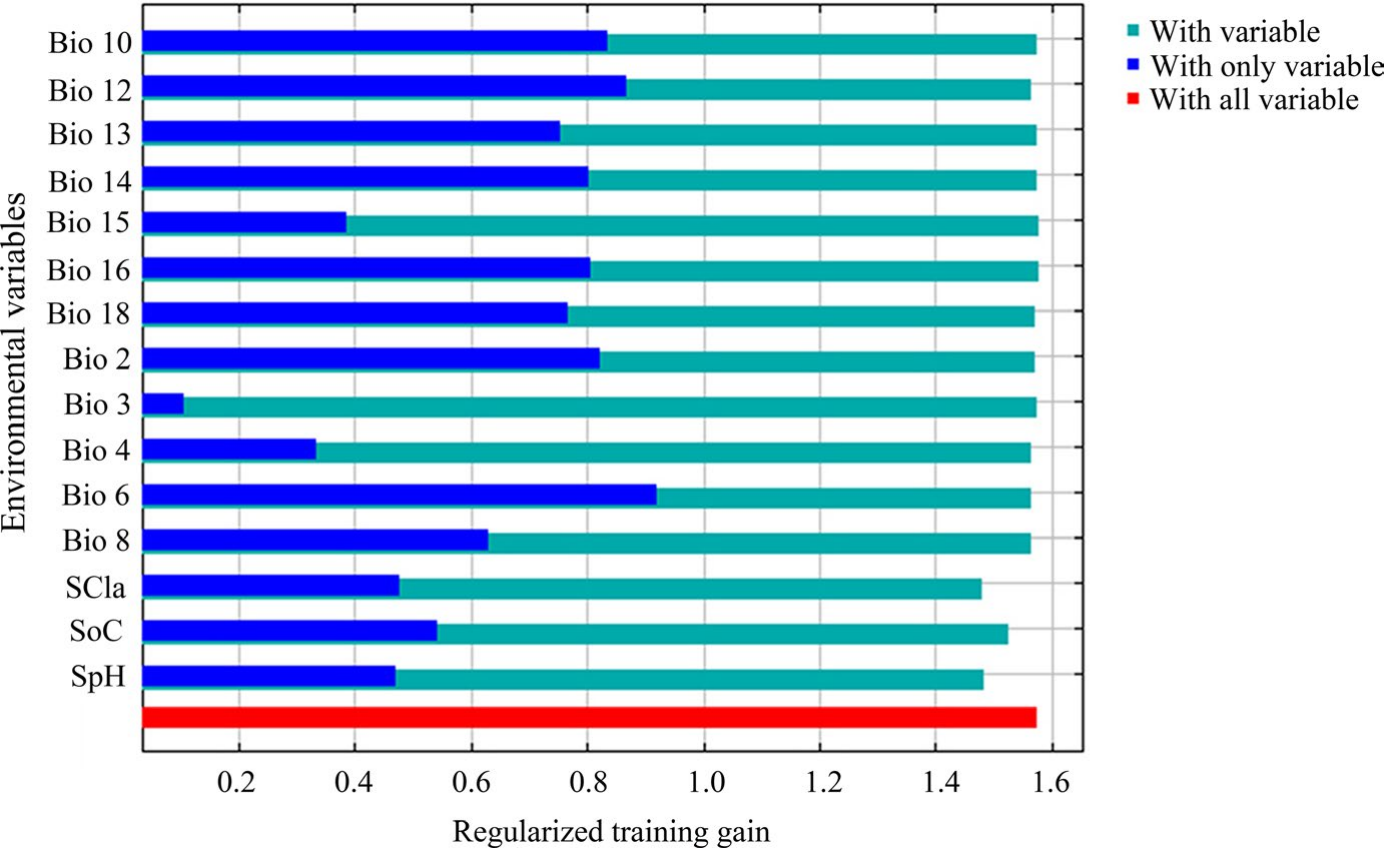
Jackknife test for evaluating the relative importance of environmental variables for *Pterocarya stenoptera* in China

According to the internal jackknife test results of the Maxent model, the factors that contribute to the distribution model for *P. stenoptera* included Bio6 (9.6% of variation), Bio10 (14.1% of variation), Bio12 (23.2% of variation) and Bio14 (29.2% of variation) (Figure 3; Table 2). These factors had the cumulative contributions up to 76.1% (Table 2). In contrast, the contributions of other variables were low in weights, suggesting that their influence on the suitable habitat distribution of *P. stenoptera* was limited (Table 2).

**TABLE 2:** Percentage contributions of the bioclimatic variables included in the Maxent models for *Pterocarya stenoptera*

Variable	Percent contribution (%)
Precipitation of driest month (Bio14)	29.2
Annual precipitation (Bio12)	23.2
Mean temperature of warmest quarter (Bio10)	14.1
Min temperature of coldest month (Bio6)	9.6
Soil pH (SpH)	8.7
Soil class (SCl)	7.9
Soil organic carbon (SC)	3.7
Mean temperature of wettest quarter (Bio8)	1.9
Precipitation of wettest month (Bio13)	0.5
Mean diurnal range (Bio2)	0.3
Isothermality (Bio3)	0.3
Temperature seasonality (Bio4)	0.2
Precipitation of warmest quarter (Bio18)	0.2
Precipitation of wettest quarter (Bio16)	0.1
Precipitation seasonality (Bio15)	0.1

**FIGURE 4:**
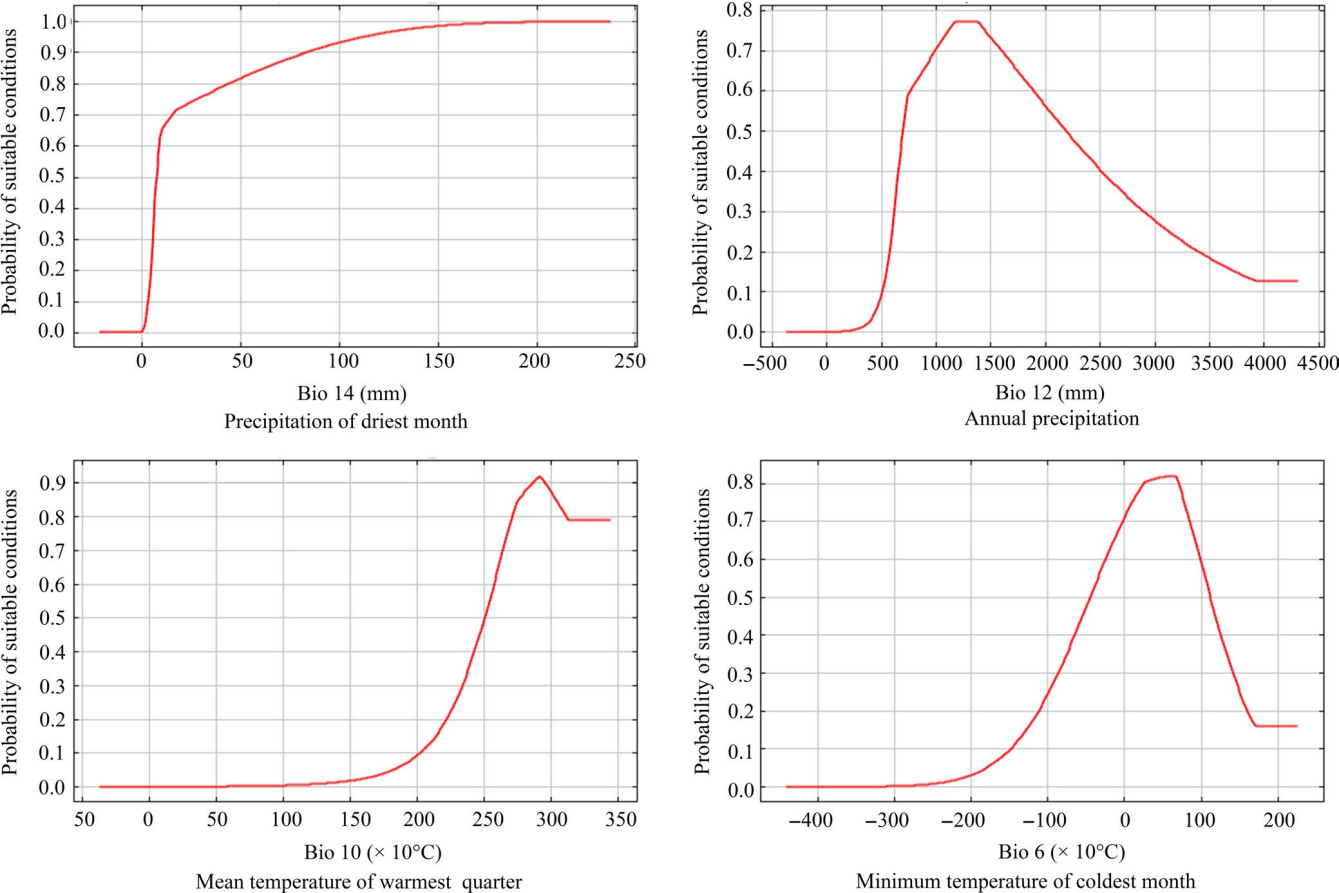
Response curves for important environmental predictors in the species distribution model for *Pterocarya stenoptera*

The individual response curves (marginal responses created by holding all other bioclimatic variables to their average sample values) for those four most important variables in Maxent were generated to examine the climatic preference of *P. stenoptera* (Figure 4). Overall, there was a positive nonlinear response observed for the precipitation in the driest month (Bio14). The optimum annual precipitation (Bio12), minimum temperature in the coldest month (Bio6), and average temperature in the warmest quarter (Bio10) were approximately 1,300 mm, 5°C, and 28°C.

### 3.3 Current potential distribution of *P. stenoptera*

**FIGURE 5:**
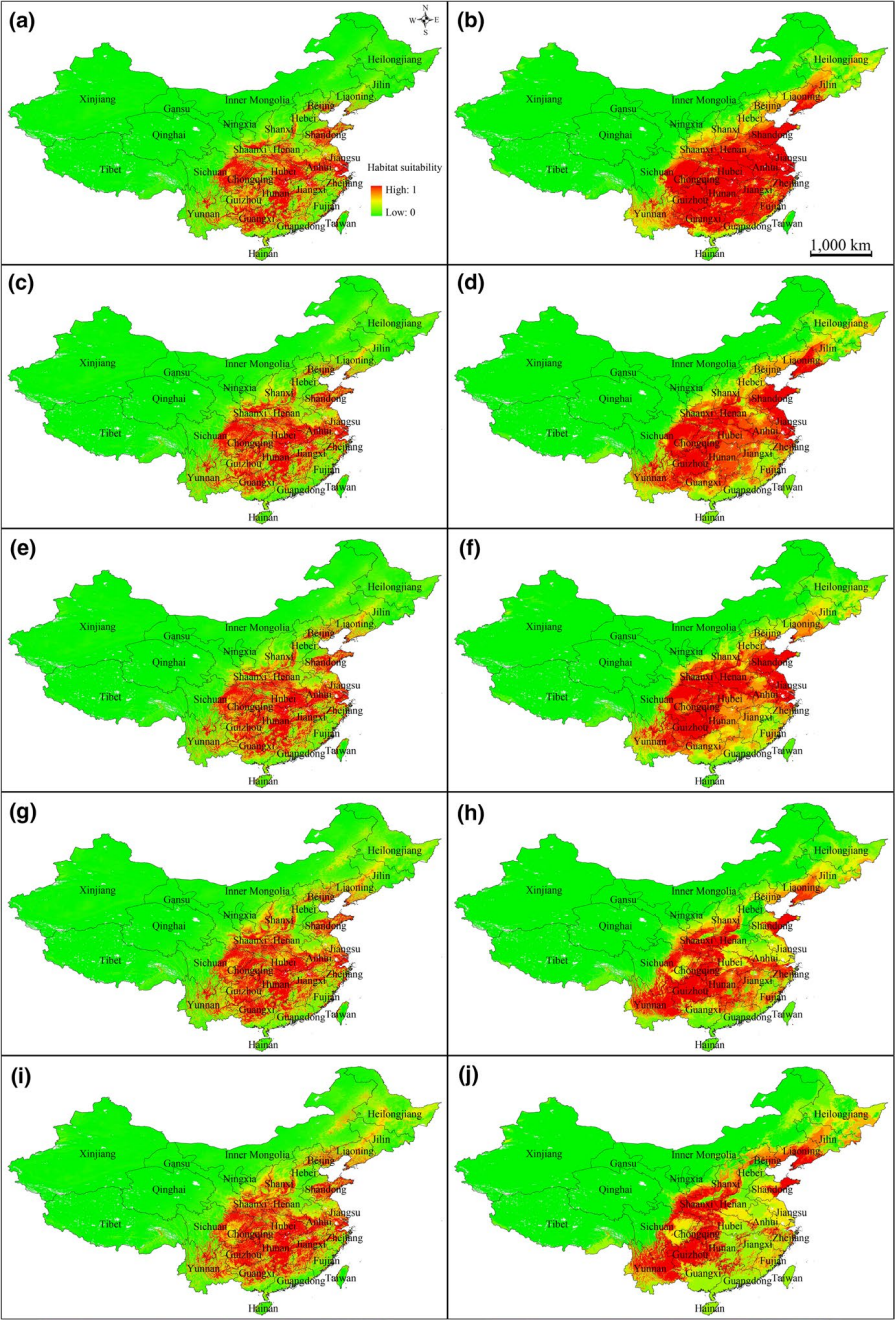
Predicted potential distribution map of *Pterocarya stenoptera* using Maxent (a, c, e, d, and i) and GARP (b, d, f, h, and j). a and b, current climate scenario; c and d, future climate scenario RCP 2.6 in 2050; e and f, future climate scenario RCP 2.6 in 2070; g and h, future climate scenario RCP 8.5 in 2050; i and j, future climate scenario RCP 8.5 in 2070 (nine‐dashed line in South China Sea not shown)

According to the prediction results of Maxent and GARP models, the climate in most coastal regions of southern China, such as Guizhou, Guangxi, Guangdong, Fujian, Hubei, Jiangxi, Zhejiang, Anhui, Jiangsu, Henan, Shandong, Liaoning, eastern Sichuan, and Chongqing, is suitable for the growth of *P. stenoptera* (Figure 5). However, the current potential distributions predicted by the two algorithms are inconsistent: GARP predicted large areas in Heilongjiang, Jilin, and Hebei to be suitable, which were marginally predicted by the Maxent. Also, GARP predicted that the potential geographic distribution with high suitability was continuous and covers a large area, whereas that predicted by Maxent was scattered and small.

### 3.4 Forecasting the future distribution of *P. stenoptera*

The effects of climate change on the potential distribution of *P. stenoptera* were visually analyzed by using both emission scenarios (RCP 2.6 and RCP 8.5) and modeling methods (GARP and Maxent), showing that the climatic suitability of the existing distribution range increased, and the suitable habitats of the species expanded geographically to the north and to higher elevations (Figure 5). In the two emission scenarios by 2070, both GARP and Maxent predicted that the areas suitable for the species decreased in Taiwan, Hainan, and the southern limit of the species' distribution and increased in northeast Inner Mongolia, Heilongjiang, and Jilin. On average, Maxent predicted the suitable habitat conditions for China will increase by 4.51% and 9.49% by 2070 under RCP 2.6 and RCP 8.5, respectively. However, GARP predicted the suitable habitat conditions for China will decline by 0.54% and increase 2.4% by 2070 under RCP 2.6 and RCP 8.5, respectively (Figure 5).

## 4 DISCUSSION

One prerequisite for using a species in ecosystem restoration is gaining a detailed understand of its distribution. *P. stenoptera* is a common fast‐growing tree species often used in the ecological restoration of riverbanks and alpine forests in central and eastern China. However, the impacts of climate changes on its distributions of have not been examined. In the present study, we model the distributions of *P. stenoptera* under current and future climate scenarios.

### 4.1 Model performance

Previous studies have shown that model performance differs among different ENMs based on the sampling size, the study area, and the species modeled (Elith et al., 2006; Hernandez et al., 2006; Pearson et al., 2006; Tsoar, Allouche, Steinitz, Rotem, & Kadmon, 2007). Because of the variation of predictions, the “optimal” modeling technique is difficult to identify (Pearson et al., 2006). Elith et al. (2006) showed that Maxent had the highest performance in a comparison of several habitat suitability models, and the AUC values were near 1.0. In contrast, the performance of GARP was poor, and the AUC values were low. However, Peterson et al. (2007) indicated that GARP was better at predicting the distributions of species that occupy broad areas than Maxent. Here, we have found that both GARP and Maxent have high AUC and TSS values and low OR values. The results from all three evaluation methods suggest both models have good predictive power. Moreover, although the AUC, TSS, and OR values of Maxent were statistically significantly higher than GARP, the size of difference is small (Table 1). Therefore, we can conclude that the performance of Maxent and GARP was roughly the same.

From the geographic perspective, GARP predicted large areas to be environmentally suitable, which were marginally predicted by the Maxent model. Also, GARP predicted the potential geographic distribution with high suitability was continuous and covers a large area, whereas those predicted by Maxent were scattered and small. Although GARP prediction was too extensive and Maxent models underestimated the species range especially in the Heilongjiang, Jilin, and Hebei, both algorithms drew maps consistent with the known distribution of the species (Figure 5). Similar results were found by Elith et al. (2006) and Hernandez et al. (2006).

### 4.2 Ecological processes that influence the distribution of *P. stenoptera*

An important issue in evolution and ecology are the factors influencing and maintaining geographic distributions (Austin, 2007; Bucklin et al., 2015; Guisan & Zimmermann, 2000). Among the 15 environmental variables adopted in the model, the most important ones that explained the species' environmental requirements best were two temperature‐derived variables and two precipitation‐derived variables, that is, precipitation of driest month (Bio14, 29.2% of variation), annual precipitation (Bio12, 23.2% of variation), mean temperature of warmest quarter (Bio10, 14.1% of variation), and minimum temperature of coldest month (Bio6, 9.6% of variation) (Figure 4; Table 2). Note that marginal responses were obtained by keeping all other bioclimatic variables at their average sample value. In reality, variables are not held at their means. There are interactions between variables that modify suitability in ways that are completely obscured by marginal response curves. Nevertheless, it allows us to see the relationships between selected variables and probability of suitable conditions.

*Pterocarya stenoptera* occurs extensively in the warm temperate and subtropical zones of China along wet hillside land or along streams. The species can tolerate high temperatures and long periods of inundation (up to 12 months) (Li et al., 2010). Drought limits plant growth when precipitation is low and results in low river levels (Pan, 2009). When comparing the effects of flood and submergence on seedlings to the effects of drought, seedlings wilted more severely during drought resulting in plant death after day 9–15 (Yang et al., 2013). The photosynthesis, respiration, and transpiration of *P. stenoptera* subjected to drought decreased (Pan, 2009). Therefore, changes of precipitation of driest month and annual precipitation will affect the distribution of *P. stenoptera* as indicated in our results. Areas with rainfall less than 50 cm annually are to be discarded because they do not provide suitable habitat for *P. stenoptera* (Pan, 2009). However, plants of *P. stenoptera* can withstand a short period of −20°C weather during the middle of winter, but a longer cold spell with temperatures of −10°C could cause serious dieback (Pan, 2009). Previous studies have shown that seedling emergence and death of *P. stenoptera* were directly affected by winter temperature in *P. stenoptera* (Wang et al., 2018). Therefore, these hydrothermal factors may influence the distribution of *P. stenoptera* by influencing the photosynthesis, respiration, transpiration, seed germination, growth and reproduction of *P. stenoptera.*

### 4.3 Impact of climate change on the distribution of *P. stenoptera* and associated forest ecosystems

Most global climate models estimate the global warming at a rate of 0.2°C per decade (Stocker et al., 2013). An increase in temperature will accelerate many physiological processes. For example, photosynthesis will reach an upper limit in plants with increased temperature although the response will vary based on the plant species. As a consequence, it can lead to local or regional species disappearance, as well as the loss of entire ecosystems or other substituents by other ecosystems (Deb et al., 2017a; Zhang et al., 2018). The models employed here demonstrate that the spatial extent of suitable climate available for *P. stenoptera* is expected to expand geographically, especially in a northerly direction. As for both emissions scenarios and algorithms in our study, the projection of our models to future climate predicted similar distributional shift trends of *P. stenoptera*. As discussed above, mean temperature of warmest quarter and temperature of coldest month were two of the most important factors affecting *P. stenoptera* distribution. A continuous rise in temperature would be beyond the temperature tolerance of *P. stenoptera*. Moreover, alterations in the temperature and precipitation regime potentially give rise to the shifts of *P. stenoptera* species phenologically, which may also indirectly affect the dependent faunal and floral species. Moreover, those changes may also have adverse effects on a number of terrestrial insects, mammals, and birds that are indirectly or directly dependent on the seeds, fruits, and flowers from *P. stenoptera* (Butt et al., 2015; Deb et al., 2017a).

### 4.4 Implications for conservation planning

As detected by our models, the potential suitable habitat of *P. stenoptera* shifted northward and to higher elevations under future climate scenarios. The plantation of *P. stenoptera* into those potential suitable habitats may serve as a preservation strategy in the presence of future climate changes (Deb, Phinn, Butt, & McAlpin, 2017b). Additionally, our results can be utilized for categorizing the *P. stenoptera* natural habitats from a lower risk to a higher one in response to the climate change, for the sake of conservation planning. For example, the *P. stenoptera* plantations can be introduced preferentially into those climatically appropriate areas; in addition, more efforts should be made to conserve their natural regeneration among the high‐risk areas in the case of future climates. The unchanged suitable habitat can be considered as an underlying refugium of climate change, which is a vital choice for conserving and protecting *P. stenoptera* forests ex situ and in situ. Moreover, *P. stenoptera* is of vital importance for ecosystem functioning. Therefore, plantation of *P. stenoptera* represents a vital management mechanism that can be used to create secondary growth forests.

### 4.5 Limitations of the modeling approach and future research directions

Ecological niche modellings have been recognized as the efficient and extensively used approach to offer related guidelines for managing forest in the presence of global climate change (Deb et al., 2017b). Nonetheless, uncertainties in using various GCMs in projecting the potential distribution exist (Wiens, Stralberg, Jongsomjit, Howell, & Snyder, 2009). In this study, the BCC‐CSM1.1 was adopted. Although this GCM has been recommended to be used in studies investigating the climate changes across China, the nature of climate change is uncertain, and hence, the projected distribution/suitability of habitat is also uncertain. Therefore, different GCMs should be used in future studies. Additionally, although GARP and Maxent models are widely used, some limitations should also be noted (Elith et al., 2006). In the present study, presence‐only data are compiled based on a variety of sources. Consequently, a greater number of species occurrence records may have been included within those relatively well‐known areas in comparison with other native ranges (Deb et al., 2017a). However, the sampling bias layer adopted within our models only represents a close estimation for actual species distribution. Current ENMs hypothesize that the species distribution observed is not impacted by the source–sink dynamics or dispersal limitations; besides, the complex transient dynamics are also ignored during the range shifting process. Therefore, species dispersal constraints should be added to models, not only see how climate change will shift the species' niche, but to see how much the species could actually shift given species dispersal limitations. However, the quantitative descriptions of dispersal constraints for the studied species were not included in the models due to the lack of dispersal and demographic parameters. Moreover, various variables that are recognized to be vital parameters (such as, soils, logging pressure, dispersal capabilities, competition, and human activities) were not incorporated within distribution modeling as a result of the insufficient robust data. Nevertheless, future studies should incorporate them for analysis.

## 5 CONCLUSIONS

Under the climate change scenario, high‐quality distributional data play an important role in setting priorities and implementing effective protection actions. Our results indicate that the areas of suitable habitat for *P. stenoptera* are projected to increase, and its climatic niche expands geographically to the north and higher elevation. Minimum temperature of coldest month (Bio6), mean temperature of warmest quarter (Bio10), annual precipitation (Bio12), and precipitation of driest month (Bio14) were the most important environmental variables influencing the distribution of this species. The projected spatial and temporal patterns of *P. stenoptera* can provide reference for the development of forest management and protection strategies.

## CONFLICT OF INTEREST

The authors declare no competing interest.

## AUTHOR CONTRIBUTIONS

**Keliang Zhang:** Formal analysis (equal); funding acquisition (equal); methodology (equal); writing – original draft (equal). **Huina Liu:** Formal analysis (equal); writing – original draft (equal); writing – review and editing (equal). **Haolei Pan:** Data curation (equal); investigation (equal); resources (equal); software (equal). **Yi Zhao:** Data curation (equal); investigation (equal); resources (equal); software (equal). **Wenhao Shi:** Data curation (equal); investigation (equal); resources (equal); software (equal); visualization (equal). **Silei Li:** Data curation (equal); investigation (equal); resources (equal); visualization (equal); writing – original draft (equal). **Junchi Liu:** Data curation (equal); investigation (equal); resources (equal); software (equal); visualization (equal). **Jun Tao:** Conceptualization (lead); formal analysis (equal); project administration (lead); supervision (lead); writing – review and editing (equal).

## Data Availability

Climate data from Worldclim database were used (worldclim.org). Sampling locations and data process are stored in Dryad repository (https://doi.org/10.5061/dryad.s7h44j13m).
